# The Emerging Role of Left Atrial Strain in Cardiovascular Risk Stratification for Multiple Myeloma Patients Undergoing Carfilzomib Therapy

**DOI:** 10.3390/cancers17142375

**Published:** 2025-07-17

**Authors:** Anna Colomba, Lorenzo Airale, Alice Lasagno, Giulia Mingrone, Anna Astarita, Fabrizio Vallelonga, Dario Leone, Martina Sanapo, Arianna Paladino, Francesca Novello, Sara Bringhen, Francesca Gay, Franco Veglio, Alberto Milan

**Affiliations:** 1 Candiolo Cancer Institute, FPO—IRCCS, 10060 Candiolo, TO, Italy; anna.colomba@unito.it (A.C.); alice.lasagno@edu.unito.it (A.L.); giulia.mingrone@unito.it (G.M.); dario.leone@ircc.it (D.L.); martina.sanapo@unito.it (M.S.); ariannapaladino@hotmail.it (A.P.); francesca.novello@unito.it (F.N.); alberto.milan@unito.it (A.M.); 2 Department of Medical Sciences, University of Turin, 10126 Turin, Italy; 3 Hypertension Unit, Department of Medical Sciences, Division of Internal Medicine, AOU Città della Salute e della Scienza University Hospital, 10126 Turin, Italy; lorenzo.airale@unito.it (L.A.); anna.astarita@unito.it (A.A.); franco.veglio@unito.it (F.V.); 4 SSD Clinical Trials in Oncoematologia e Mieloma Multiplo, Division of Oncology, AOU Città della Salute e della Scienza University Hospital, 10126 Turin, Italy; sarabringhen@yahoo.com (S.B.); francesca.gay@unito.it (F.G.); 5 Department of Molecular Biotechnology and Health Sciences, Division of Hematology, University of Turin, 10124 Turin, Italy

**Keywords:** multiple myeloma, cardiovascular risk, cardio-oncology, left atrial strain, arterial hypertension

## Abstract

Background: Carfilzomib (CFZ) is a proteasome inhibitor with known cardiotoxic effects used in multiple myeloma (MM) treatment. Cardio-oncology guidelines recommend cardiovascular risk assessment via echocardiography. Left atrial strain (LAS) is not yet included as a marker of cardiotoxicity, but it is emerging as a potential indicator of cardiac dysfunction. Objective: This study evaluates LAS as a predictor of CFZ-related hypertensive cardiovascular adverse events (CVAEs) in MM patients, with or without prior hypertension. Methods: A total of 125 MM patients treated with CFZ at the Hypertension Center, “Città della Salute e della Scienza” in Turin, were enrolled. Baseline assessments included transthoracic echocardiography for LAS analysis via Philips QLAB software. Results: During CFZ therapy, 52% of patients experienced hypertensive events. LAS conduit was significantly impaired in those who experienced CVAEs (−16.20 [−20.75; −12.65] vs. −20.80 [−26.30; −15.40], *p* = 0.006) and LAS conduit > −22 acted as a predictor of hypertensive adverse events in the normotensive population (OR 2.37 [1.02; 5.50]). Conclusion: These findings indicate that alterations in LAS conduit are linked to an increased risk of hypertensive adverse events during CFZ treatment. Incorporating LAS measurement into cardiovascular risk assessments may improve personalized risk stratification for MM patients, especially those without pre-existing hypertension.

## Simple Summary

Carfilzomib (CFZ) is a cardiotoxic drug used in multiple myeloma (MM) treatment protocols. Cardio-oncology guidelines suggest cardiovascular risk stratification via echocardiography, not yet including left atrial strain (LAS) assessment. This study explores LAS as a predictor of CFZ-induced hypertensive cardiovascular adverse events (CVAEs) in MM patients, with or without pre-existing hypertension. A cohort of 125 MM patients receiving CFZ was monitored, with 52% experiencing hypertensive events. LAS conduit, measured via Philips QLAB echocardiographic software, was significantly worse in those who experienced CVAEs (−16.20 [−20.75; −12.65] vs. −20.80 [−26.30; −15.40], *p* = 0.006). Additionally, LAS conduit > −22 predicted hypertensive adverse events in normotensive patients (OR 2.37). These results highlight the association between altered LAS parameters and increased hypertensive risk during CFZ therapy. Integrating LAS into current cardiovascular evaluations could improve risk stratification and allow more personalized management of MM patients, particularly those without pre-existing hypertension.

## 1. Introduction

Multiple myeloma (MM) is a hematologic malignancy defined by the uncontrolled proliferation of monoclonal plasma cells, leading to excessive protein production and deposition. Cardiovascular complications are frequently observed in MM patients due to factors such as blood hyperviscosity, anemia, and light chain cardiac amyloidosis, which can ultimately contribute to the development of heart failure [1], further increasing susceptibility to cardiovascular events. Additionally, MM predominantly affects an older population with a high burden of cardiovascular comorbidities [2]. Beyond disease-related mechanisms, the cardiotoxicity of several MM treatment regimens further exacerbates cardiovascular risk [3]. Carfilzomib, a second-generation proteasome inhibitor widely used both in the relapsed/refractory setting and as a first-line therapy, has been strongly associated with cardiovascular adverse events (CVAEs) [4]. The underlying mechanisms of Carfilzomib-induced cardiotoxicity remain incompletely understood; however, myocardial cells appear particularly vulnerable to proteasome inhibition given the critical involvement of the ubiquitin–proteasome system in maintaining intracellular metabolic homeostasis [5]. In addition, blocking proteasomal activity has been associated with decreased endothelial nitric oxide synthase (eNOS) function, resulting in diminished nitric oxide (NO) bioavailability and consequent arterial hypertension [6], which occurs in approximately 12% of patients during treatment [7]. Given these risks, precise cardiovascular (CV) risk stratification before initiating Carfilzomib therapy is of critical importance.

The most recent cardio-oncology recommendations [8] identify echocardiography as the preferred initial imaging technique for evaluating cardiac performance, particularly through the measurement of left ventricular ejection fraction (LVEF) and left ventricular global longitudinal strain (LV GLS). While not commonly part of the standard baseline cardiovascular workup, left atrial strain (LAS) has gained attention as a sensitive marker of early cardiac dysfunction. Emerging data indicate that LAS could hold prognostic value in patients with heart failure with preserved ejection fraction (HFpEF) [9] and has demonstrated utility in assessing diastolic dysfunction, as it can identify alterations in ventricular filling pressures preceding overt structural and functional changes [10]. Furthermore, recent studies have explored its prognostic significance in oncology patients undergoing chemotherapy [11,12]; however, its role in MM patients receiving Carfilzomib remains uninvestigated.

**Figure 1:**
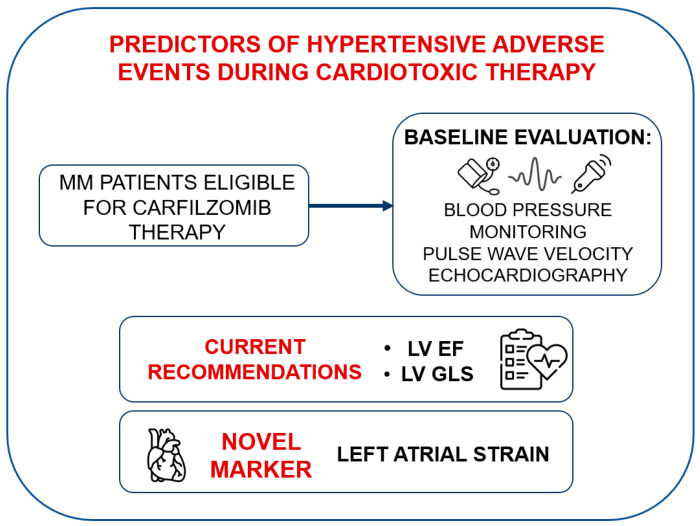
Clinical workflow involving left atrial strain. MM: multiple myeloma, LV: left ventricular, EF: ejection fraction, GLS: global longitudinal strain.

This study aims to evaluate the role of LAS, a parameter of growing interest due to recent evidence of its sensitivity in detecting early cardiac dysfunction, as a predictive marker for Carfilzomib-related hypertensive CVAEs in MM patients, with the objective of improving baseline cardiovascular risk stratification in this population (Figure 1).

## 2. Materials and Methods

This single-center investigation took place at the Hypertension Unit and Cardiovascular Diseases Center of “Città della Salute e della Scienza” Hospital in Turin, Italy. Ethical approval was obtained from the institutional bioethics committee of the “A.O.U. Città della Salute e della Scienza” hospital, Turin, Italy (protocol number 0038655). All enrolled participants were aged >18 years and provided written informed consent in accordance with the principles of the Declaration of Helsinki. The study included oncologic patients diagnosed with multiple myeloma who were all referred by hematologists to the Hypertension Unit for cardiovascular risk evaluation prior to starting Carfilzomib-based therapy, with enrollment taking place between January 2015 and March 2023.

Exclusion criteria consisted of prior exposure to cardiotoxic agents, the presence of light chain amyloidosis and the absence of informed consent. Those with poor-quality echocardiographic images were also excluded due to the inability to calculate LAS.

### 2.1. Clinical Assessment

All patients underwent a thorough initial evaluation following ESC/ESH [13,14] and European Myeloma Network [15,16] recommendations, including cardiovascular and oncologic history, office and ambulatory blood pressure monitoring (ABPM), 12-lead electrocardiogram (ECG), pulse wave velocity (PWV) measurement for arterial stiffness assessment, transthoracic echocardiography (TTE), and SCORE-2 risk estimation based on age, sex, smoking status, systolic blood pressure, and total cholesterol, as outlined by ESC guidelines [14]. Hypertensive status was defined based on a documented history of arterial hypertension or a new diagnosis established through ABPM. Arterial stiffness was qualified using PWV, measured with a validated device (Sphygmocor System Atcor Medical, Sydney, Australia). A PWV value exceeding 9 m/s was considered indicative of subclinical vascular organ damage [17]. Transthoracic echocardiograms were performed by skilled operators certified by the European Association of Cardiovascular Imaging (EACVI). An average of three cardiac cycles per measurement were acquired. Left ventricular (LV) mass and structural parameters were calculated using the Devereux formula and indexed to body surface area and height raised to the 2.7 power (LVMi); LV hypertrophy (LVH) was defined by LVMi thresholds of ≥115 g/m^2^ (≥49 g/m^2.7^) for men and ≥95 g/m^2^ (≥47 g/m^2.7^) for women. LV ejection fraction (LVEF) was derived from apical four- and two-chamber views, while global longitudinal strain (GLS) was measured using speckle-tracking echocardiography with the Automated Cardiac Motion Quantification software (QLAB Cardiac Analysis v15, Philips, Andover, MA, USA) in accordance with established protocols [18]. A more comprehensive description of our methodology is provided in our recent publication [19].

### 2.2. Left Atrial Strain

**Figure 2:**
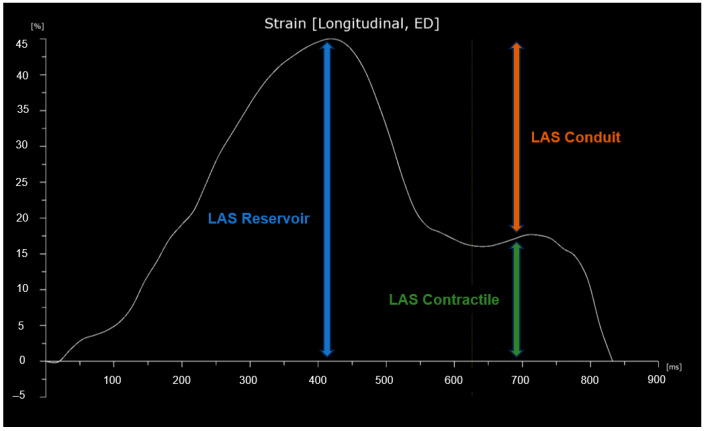
Graphical representation of left atrial strain (LAS). The curve begins at the onset of the QRS complex.

Left atrial strain was evaluated using apical four-chamber views obtained with speckle tracking echocardiography (STE). The images were optimized for orientation, depth, and gain to prevent foreshortening of the LA and to visualize the entire LA throughout the cardiac cycle. The recordings were analyzed using specialized software (Automated Cardiac Motion Quantification, QLAB Cardiac Analysis, Philips, Andover, MA, USA) enabling offline semi-automated evaluation of speckle-based strain. The tracking was manually adjusted by the operator when necessary, excluding the LA appendage and the pulmonary vein openings [20]. Left atrial strain was measured in accordance with EACVI recommendations, using the onset of the QRS complex on the ECG as the temporal reference point [20,21]. LAS reservoir (LAS-r) was derived from the strain curve as the peak value, LAS contractile (LAS-ct) as the strain value at the onset of atrial contraction, and LAS conduit (LAS-cd) as the difference between LAS-r and LAS-ct. A graphical representation of LAS is shown in Figure 2.

### 2.3. Statistical Analysis

Statistical analyses were performed using dedicated software (R: A Language and Environment for Statistical Computing, version 4.3.2 for Mac OSX, R Core Team, Vienna, Austria; and Jamovi, version 2.2.5). The distribution of continuous variables was assessed with the Shapiro–Wilk test; results are presented as mean ± standard deviation or median [interquartile range] for continuous data, and as counts and percentages for categorical data. Between-group differences were analyzed using the Student’s *t*-test or Wilcoxon rank-sum test for continuous variables, and the Pearson chi-square or Fisher’s exact test for categorical variables. Survival analysis was carried out using both univariate and multivariate Cox proportional hazards models, with the proportional hazards assumption assessed via the Schoenfeld residuals test.

Covariates for inclusion in the multivariate models were selected from those identified as significant in the univariate analysis. These variables were incorporated into a model that evaluated all possible combinations and selected the best-fitting models based on AIC metrics. Thresholds for dichotomizing continuous variables were determined using the maximally selected rank statistic. A *p*-value < 0.050 for two-tailed tests was considered statistically significant for all analyses.

## 3. Results

Out of 156 patients with MM, 125 patients were included in the following analyses (see the flowchart of the study population in Figure S1 in Supplementary Materials).

### 3.1. General Characteristics and Cardiovascular Risk Factors

Patients had a median age of 67.7 ± 8.71. Arterial hypertension was the most represented cardiovascular comorbidity, with a prevalence of 34.4%. Patients who experienced hypertensive adverse events were exposed to higher cardiovascular risk, as documented by statistically significant higher SCORE-2 (13.0% [6.80; 17.6] vs. 7.95% [4.85; 11.0], *p =* 0.029), PWV values (8.75 [7.31; 9.90] vs. 7.25 [6.42; 8.15], *p* = 0.002), and LVH prevalence (43.07% vs. 23.33%, *p* = 0.018) compared with patients without hypertensive adverse events during treatment. There were no significant differences in terms of left atrial dimension of function, except for LAS-cd, significantly reduced in patients with hypertensive adverse events (−16.20 [−20.75; −12.65] vs. −20.80 [−26.30; −15.40], *p* = 0.006). Results are presented in Table 1. See Table S1 in Supplementary Materials for general characteristics of hypertensive vs. normotensive populations.

### 3.2. Cardiovascular Hypertensive Adverse Events

Half of the study population (52%, 65 patients) experienced hypertensive adverse events during treatment with Carfilzomib, the most common being the worsening of pre-existing arterial hypertension (48.38% of total population, 60 patients). Non-target blood pressure values were observed in 41 patients prior to drug infusion, leading to non-administration in 12 cases (9.6%). After Carfilzomib infusion, 18 patients (14.4%) presented suboptimal blood pressure values. Hypertensive urgencies occurred in 5 patients (4% of cases). A summary of these events is presented in Table 2.

### 3.3. Predictive Variables of Hypertensive Events

Continuous variable and dichotomized variable models were tested for prediction of hypertensive adverse events during Carfilzomib therapy. The analysis of dichotomic variables demonstrated that SBP > 122 mmHg, BPV > 9, and standard deviation Daytime blood pressure (DS_day) > 8 mmHg on ABPM were significantly associated with an increased risk of hypertensive events (*p* < 0.008). Additionally, PWV > 9 m/s was also identified as a significant predictor (*p* = 0.004). In terms of echocardiographic variables, left atrial volume index (LAVi) > 37 mL/m^2^ (*p* = 0.047), ascending aorta diameter (AO ASC) > 35 mm (*p* = 0.046), and LAS conduit > −22% (*p* < 0.006) were significantly associated with hypertensive events (Table 3).

When these variables with statistically significant predictive value were incorporated into a multivariate model, SBP > 122 mmHg, DS_day > 8 mmHg and BPV > 9 on ABPM, PWV > 9 m/s, ascending aorta diameter > 35 mm, LAVi > 37 mL/m^2^, and LAS conduit > −22% emerged as the most influential covariates in predicting the likelihood of developing hypertensive events, with statistical significance reached only for LAS conduit > −22% (Table 4).

**Figure 3:**
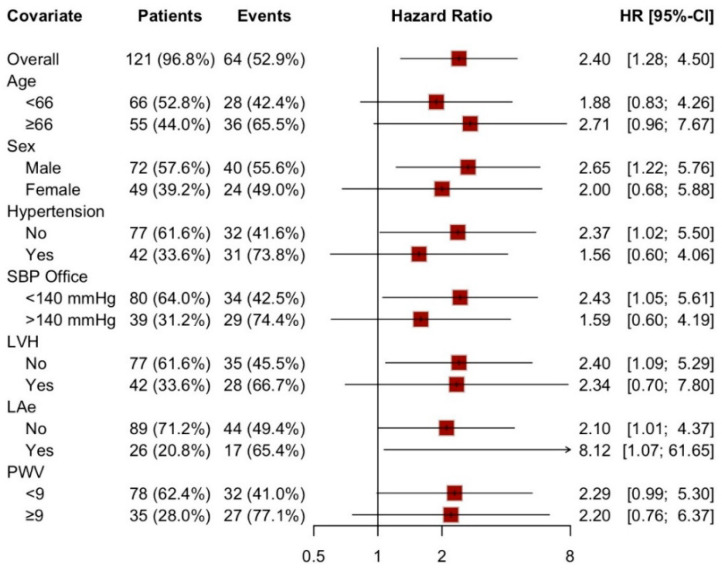
Forest plot: LAS-cd > −22% as a predictive factor in different sub-population. SBP: systolic blood pressure, LVH: left ventricular hypertrophy, LAe: left atrial enlargement, PWV: pulse wave velocity.

Notably, LAS conduit > −22% demonstrated a consistent predictive trend for hypertensive events even after stratifying the population into subgroups based on various anthropometric and echocardiographic characteristics, as depicted in Figure 3. In this stratified analysis, statistical significance was preserved after adjusting for male sex, history of normotension, PAS < 140 mmHg, and the absence of left ventricular hypertrophy (LVH) or left atrial enlargement (LAe).

Continuous variable results are summarized in Supplementary Materials Tables S2 and S3 and Figure S2.

## 4. Discussion

In this retrospective analysis, we explored the potential role of left atrial strain in predicting hypertensive events during cardiotoxic therapy. To the best of our knowledge, this is the first work implementing left atrial strain in baseline cardiovascular risk stratification for multiple myeloma patients treated with Carfilzomib.

The left atrium is a dynamic structure that performs multiple functions throughout the cardiac cycle: it serves as a reservoir during systole and contributes to filling the left ventricle in diastole, both by following the pressure gradient and, in patients without atrial fibrillation, through active contraction. Due to its anatomy, the left atrium is deeply influenced by the neighboring structures. Its posterior wall remains relatively fixed throughout the cardiac cycle as it is anchored by the pulmonary veins [22]. Therefore, atrial deformation largely depends on the excursion of the atrioventricular plane. Moreover, given that the LV apex remains essentially stationary throughout the cardiac cycle, longitudinal shortening of the LV induces stretching of the LA [23]. This physiological interplay elucidates how LA compliance (reservoir function) is modulated by LV systolic function and GLS via atrioventricular plane displacement [24], while the conduit function is primarily determined by LV diastolic function, relying on both the intrinsic stiffness of the LV chamber and the suction force generated by ventricular relaxation; the contractile phase is influenced by inherent LA contractility as well as LV end-diastolic filling pressures [10].

The existing literature primarily focuses on the reservoir and contractile phases [20]. More precisely, LAS-r has demonstrated promising prognostic value in patients with EF-preserved heart failure and diastolic disfunction, due to its correlation with filling pressures [24,25]. In fact, LAS-r and LAS-ct have been proposed as additional parameters for evaluating indeterminate LV filling pressures when standard parameters in the EACVI/ASE flow chart are inconclusive [23,26]. In the oncologic population, LAS-r has been investigated as a marker for cardiac injury during chemotherapy with anthracyclines [27], and it has been preliminarily proposed as a predictor of cardiotoxicity in addition to ventricular GLS [11].

Conduit strain is derived from the difference between reservoir and conduit values, using the onset of the QRS complex on the electrocardiogram as the reference point. Although it is less frequently reported in the literature, its prognostic value has recently been investigated via CMR in patients with dilated and hypertrophic cardiomyopathies [28,29] and as a marker of reverse remodeling in non-ischemic cardiomyopathy [30]. However, its definitive role in a cardio-oncology setting remains uncertain, particularly in cardiovascular risk stratification prior to starting cardiotoxic therapy.

The present study complied with the cardiovascular risk stratification guidelines developed by the European Hematology Association and the European Myeloma Network [15,16] as previously described in our earlier research [31]. Applying this protocol, we confirmed the elevated cardiovascular risk of MM patients, reflected by a high frequency of cardiovascular risk factors and comorbid conditions. Additionally, the significant presence of vascular and cardiac subclinical organ damage was demonstrated by 31% of patients exhibiting arterial stiffness (PWV > 9 m/s) and 34% showing signs of cardiac remodeling (LVH). These findings further emphasize the importance of accurate baseline cardiovascular risk stratification prior to the initiation of cardiotoxic drugs, such as Carfilzomib, which is known for its cardiac and vascular toxicity [32]: notably, half of our study cohort (52%) experienced hypertensive events during treatment.

Inclusion of LAS in this baseline cardiovascular evaluation provided valuable information. Specifically, LAS-cd > −22% was associated with an increased risk of developing hypertensive adverse events during treatment. Unlike other predictive variables identified in the univariate model, such as SBP > 140 mmHg, PWV > 9 m/s, and echocardiographic variables like LAVi > 37 mL/m^2^, LAS-cd was the sole variable to demonstrate statistical significance in the multivariate model. Furthermore, after stratifying our study cohort into various subgroups, we observed a statistically significant role of LAS-cd in different subpopulations: male sex, normotension, and absence of LVH. A borderline association (CI 0.99–5.3) was also noted for patients with PWV < 9 m/s. These findings highlight the potential role of LAS as a prognostic marker for hypertensive adverse events, particularly in a lower cardiovascular risk population, meaning patients without hypertension and with neither ventricular nor vascular remodeling. Moreover, LAS is a reproducible, cost-effective technique that can be easily performed in any center equipped with speckle-tracking echocardiography, making it an accessible and valuable tool for cardiovascular screening in oncology patients.

Given the close coupling between the left atrium and ventricle, a recent study [12] conducted on MM patients suggested LAS as an additional parameter to LV GLS, although it was independently associated with survival. Based on our findings, we propose a similar application of atrial strain specifically in the lower-risk population, as the cardiovascular profile of high-risk patients is sufficiently explained by standard markers of subclinical organ damage. Its implementation in the lower cardiovascular risk population could be valuable for identifying patients at higher susceptibility to hypertensive adverse events, with the ultimate goal of enabling a more personalized follow-up strategy.

### Study Limitation

Our study has several limitations. First, the retrospective design limits the ability to establish causality between LAS and hypertensive events, necessitating prospective validation. The absence of a matched control group may have limited the strength of our findings. Additionally, since it is a single-center study, the findings may not be generalizable to larger populations of MM patients. The inclusion criteria also narrow the applicability of the results to the wider MM population, particularly for patients with amyloidosis or prior cardiotoxic treatments. Additionally, a considerable number of subjects had to be excluded due to inadequate echocardiographic image quality, which notably reduced the final sample size. Furthermore, the limited number of participants did not allow for an in-depth evaluation of hematologic variables such as ISS stage and cytogenetic risk classification. Finally, our results in the high-risk group may be limited by the sample size. It is possible that in larger cohorts, LAS could still provide incremental value and help refine risk stratification even in this subgroup. In light of these constraints, additional research is necessary to deepen our understanding of cardiovascular risk profiles in oncology patients.

## 5. Conclusions

This study emphasizes the potential of LAS, particularly LAS-cd, as a predictor of hypertensive events during Carfilzomib therapy in MM patients, with a stronger predictive value observed in lower cardiovascular risk populations. Integrating LAS into baseline cardiovascular assessments could improve risk stratification, facilitate a more tailored follow-up approach, and support early decisions to reduce or discontinue cardiotoxic therapy before irreversible damage occurs.

## Figures and Tables

**Table 1:** General characteristics of total population, patients with and without hypertensive adverse events during treatment.

General Characteristics	Total Population (n: 125)	Event (n: 65)	No Event (n: 60)	*p*-Value (Event vs. No Event)
Age, years	67.7 ± 8.71	68.8 ± 9.35	66.6 ± 7.87	0.145
Female sex, n (%)	52 (65)	24 (36.9%)	28 (46.7%)	0.356
BSA, m^2^	1.79 ± 0.21	1.81 ± 0.21	1.77 ± 0.21	0.254
BMI, Kg/m^2^	27.3 ± 4.50	27.2 ± 4.95	27.3 ± 4.00	0.889
SBP, mmHg	129 ± 18.1	135 ± 16.1	122 ± 17.7	**<0.001**
DBP, mmHg	76.3 ± 11.6	78.8 ± 11.2	73.6 ± 11.6	**0.012**
Arterial Hypertension, n (%)	42 (34.4)	31 (48.4%)	11 (19.0%)	**0.001**
Diabetes, n (%)	10 (8.06)	4 (6.15%)	6 (10.2%)	0.516
Chronic Ischemic Heart Disease, n (%)	3 (2.42)	1 (1.92)	2 (2.74)	0.760
Dyslipidemia, n (%)	19 (15.3)	7 (10.8%)	12 (20.3%)	0.219
Atrial Fibrillation, n (%)	3 (2.42)	2 (3.08%)	1 (1.69%)	1.000
Smoke status, n (%)	65 (52.4)	37 (56.9%)	28 (47.5%)	0.140
SCORE-2, %	8.50 [6.00; 14.1]	13.0 [6.80; 17.6]	7.95 [4.85; 11.0]	**0.029**
DS day, mmHg	10.4 [8.76; 13.2]	11.0 [9.59; 14.9]	9.81 [7.66; 11.4]	**0.002**
BPV	9.00 [7.00; 10.0]	9.00 [7.00; 12.0]	8.00 [6.00; 9.00]	**0.002**
PWV, m/s	7.80 [6.82; 9.36]	8.75 [7.31; 9.90]	7.25 [6.42; 8.15]	**0.002**
PWV > 9, n (%)	36 (31.3%)	28 (46.7%)	8 (14.5%)	**<0.001**
AO ASC, mm	33.6 ± 4.66	34.1 ± 3.55	33.1 ± 5.63	0.302
LVM, g	161 ± 48.3	173 ± 50.4	147 ± 42.5	**0.003**
LVMi, g/m^2^	89.8 ± 24.3	96.1 ± 26.7	83.1 ± 19.4	**0.002**
LVH, %	42 (34.1%)	28 (43.07)	14 (23.33)	**0.018**
LVEDV, mL	90.1 ± 28.0	90.3 ± 26.2	89.8 ± 30.0	0.909
LVEDVi, mL/m^2^	50.4 ± 14.3	50.1 ± 13.4	50.7 ± 15.3	0.802
LVEF, %	62.3 ± 5.92	62.4 ± 6.15	62.2 ± 5.70	0.850
GLS, %	−21.83 ± 2.51	−21.39 ± 2.34	−22.32 ± 2.62	0.053
GLS < 20, n (%)	24 (21.6%)	16 (27.1%)	8 (15.4%)	0.205
TAPSE, mm	24.0 [21.1; 27.1]	24.6 [21.1; 26.9]	23.5 [21.0; 27.1]	0.417
Diastolic Disfunction, n (%)	9 (8.26%)	5 (6.85%)	4 (5.48%)	0.704
LAS-r, %	39.5 ± 11.7	37.8 ± 12.2	41.4 ± 11.0	0.092
LAS-cd, %	−17.90 [−23.60; −13.50]	−16.20 [−20.75; −12.65]	−20.80 [−26.30; −15.40]	**0.006**
LAS-ct, %	−19.70 [−25.50; −15.70]	−19.45 [−25.30; −16.27]	−20.00 [−25.50; −15.20]	0.975
LAVi, mL/m^2^	29.0 ± 8.77	29.6 ± 8.95	28.4 ± 8.60	0.470
LAe, n (%)	29 (24.4%)	18 (29.0%)	11 (19.3%)	0.307

BSA: body surface area, BMI: body mass index, SBP: systolic blood pressure, DBP: diastolic blood pressure, SCORE-2: systematic coronary risk evaluation, DS day: standard deviation daytime blood pressure, BPV: blood pressure variability, PWV: pulse wave velocity, AO ASC: ascending aorta diameter, LVM: left ventricular mass, LVMi: left ventricular mass index, LVH: left ventricular hypertrophy, LVEDV: left ventricular end-diastolic volume, LVEDVi: left ventricular end-diastolic volume index, LVEF: left ventricular ejection fraction, GLS: global longitudinal strain, TAPSE: tricuspid annular plane systolic excursion, LAS: left atrial strain, LAS-r: LAS reservoir, LAS-cd: LAS conduit, LAS-ct: LAS contraction, LAVi: left Atrial volume index, LAe: left atrial enlargement.

**Table 2:** Hypertensive Adverse Events during Carfilzomib Therapy.

Hypertensive Adverse Events	Population, n. 125
Total Hypertensive Events, n (%)	65 (52)
Worsening of Chronic Hypertension, n (%)	60 (48.38)
Non-target blood pressure before Carfilzomib infusion (followed by infusion), n (%)	29 (23.2)
Non-target blood pressure before Carfilzomib infusion (not followed by infusion), n (%)	12 (9.6)
Non-target blood pressure after Carfilzomib infusion, n (%)	18 (14.4)
Hypertensive Urgency, n (%)	5 (4)
Hypertensive Emergency, n (%)	0 (0)

**Table 3:** Dichotomic variables: univariate analysis.

Covariate	Beta	HR (95% CI for HR)	*p* Value	Cox Assumption
Age	0.02	1.02 (0.99–1.05)	0.219	0.150
Male sex	0.23	1.26 (0.76–2.08)	0.376	0.203
SBP, (>122 mmHg)	1.32	3.75 (2.04–6.92)	**<0.001**	0.440
DS day (>8 mmHg)	1.23	3.44 (1.37–8.60)	**0.008**	0.857
BPV (>9)	0.74	2.11 (1.24–3.59)	**0.006**	<0.001
PWV (>9 m/s)	0.75	2.12 (1.26–3.54)	**0.004**	0.046
AO ASC (>35 mm)	0.58	1.79 (1.01–3.18)	**0.046**	0.080
LVEDVi (>62 mL/m^2^)	−0.54	0.59 (0.28–1.23)	0.158	0.398
GLS (>23%)	0.23	1.26 (0.70–2.27)	0.441	0.382
LAVi (>37 mL/m^2^)	0.59	1.81 (1.01–3.23)	**0.047**	0.086
LAS-cd (>−22%)	0.88	2.40 (1.28–4.51)	**0.006**	0.582

SBP: systolic blood pressure, DS: standard deviation, BPV: blood pressure variability, PWV: pulse wave velocity, AO ASC: ascending aorta diameter, LVEDVi: left ventricular end-diastolic volume index, GLS: global longitudinal strain, LAVi: left atrial volume index, LAS-cd: left atrial strain conduit.

**Table 4:** Dichotomic variables: multivariate analysis.

Covariate	Beta	HR (95% CI for HR)	*p* Value
SBP (>122 mmHg)	0.70	2.01 (0.72–5.61)	0.185
DS day (>8 mmHg)	0.81	2.24 (0.60–8.35)	0.228
BPV (>9)	0.14	1.15 (0.54–2.46)	0.710
PWV (>9 m/s)	0.08	1.08 (0.50–2.33)	0.846
AO_asc (>35 mm)	0.14	1.15 (0.56–2.36)	0.696
LAVi (>37 mL/m^2^)	0.06	1.06 (0.47–2.41)	0.887
LAS-cd (>−22%)	1.97	7.16 (1.66–30.88)	**0.008**

SBP: systolic blood pressure, DS: standard deviation, BPV: blood pressure variability, PWV: pulse wave velocity, AO ASC: ascending aorta diameter, LAVi: left atrial volume index, LAS-cd: left atrial strain conduit.

## Data Availability

The data presented in this study are available in this paper.

## Supplementary Materials

The following supporting information can be downloaded at: https://www.mdpi.com/article/10.3390/cancers17142375/s1, Figure S1: Flowchart of the study population. MM: multiple myeloma, CFZ: Carfilzomib, ABPM: ambulatory blood pressure monitoring, ECG: electrocardiogram, PWV: pulse wave velocity, TTE: trans thoracic echocardiography, LAS: left atrial strain, CV: cardiovascular. Figure S2: Forest plot: LAS-cd as a predictive factor in different sub-populations. SBP: systolic blood pressure, LVH: left ventricular hypertrophy, LAe: left atrial enlargement, PWV: pulse wave velocity. Table S1. General Characteristics of Hypertensive vs. Normotensive populations. Table S2. Continuous variables: univariate analysis. Table S3. Continuous variables: multivariate analysis.
