# Supplementary material for: The Emerging Role of Left Atrial Strain in Cardiovascular Risk Stratification for Multiple Myeloma Patients Undergoing Carfilzomib Therapy
Source: Cancers (Basel). 2025 Jul 17;17(14):2375. doi: 10.3390/cancers17142375 (PMC12294073; doi:10.3390/cancers17142375)
Supplement: Supplementary file 1 [file cancers-17-02375-s001.zip › cancers-3727376-supplementary.pdf]

Supplementary Materials

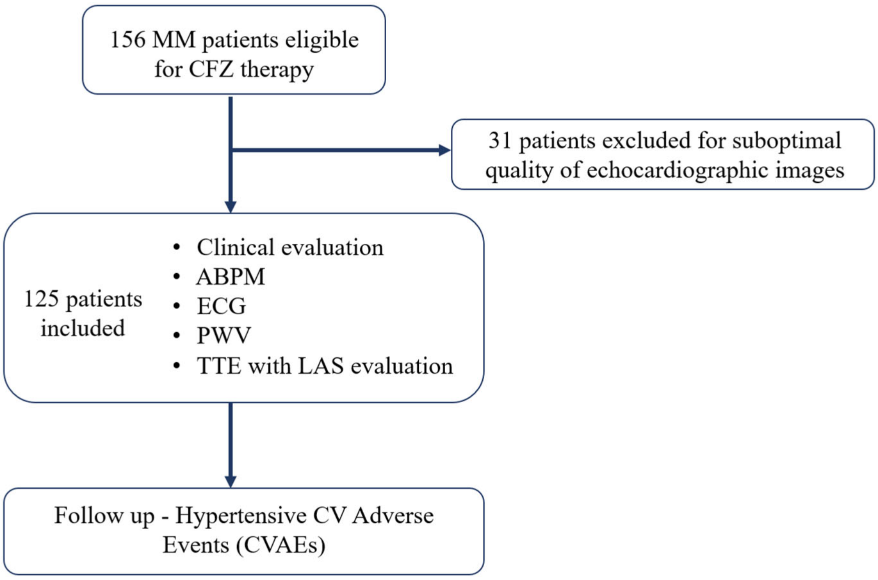

Figure S1: Flowchart of the study population. *MM: Multiple Myeloma, CFZ: Carfilzomib, ABPM: Ambulatory Blood Pressure Monitoring, ECG: Electrocardiogram, PWV: Pulse Wave Velocity, TTE: Trans Thoracic Echocardiography, LAS: Left Atrial Strain, CV: cardiovascular.*

Table S1: General Characteristics of Hypertensive vs. Normotensive populations.

General Characteristics	Normotensive (n: 47)	Hypertensive (n: 63)	p-value (Hyp vs. Norm)
Age, years	66.63±8.97	69.31±8.15	0.090
Female sex, n (%)	34 (46.6%)	18 (34.6%)	0.181
Height, cm	163.68±10.57	163.60±10.87	0.964
Weight, kg	71.18±13.03	75.98±15.84	0.066
BSA, m2	1.77±0.19	1.82±0.22	0.212
BMI, Kg/m2	26.53±4.03	28.32±4.93	0.028
Systolic Blood Pressure, mmHg	118.75±12.56	142.76±15.06	< 0.001
Diastolic Blood Pressure, mmHg	72.07±10.39	82.24±10.76	< 0.001
Diabetes, n (%)	4 (5.48%)	4 (7.69%)	0.763
Chronic Ischemic Heart Disease, n (%)	2 (2.74%)	1 (1.92%)	0.760
History of Stroke, n (%)	3 (4.11%)	0	0.136
Dyslipidemia, n (%)	10 (13.7%)	9 (17.3%)	0.602

Atrial Fibrillation, n (%)	3 (4.11%)	1 (1.92%)	0.485
Smoke status, n (%)	36 (49.3%)	29 (55.8%)	0.305
Family history of CAD, n (%)	15 (20.5%)	9 (17.3%)	0.624
Family history of stroke, n (%)	9 (12.3%)	6 (11.5%)	0.871
Family history of arterial hypertension, n (%)	16 (21.9%)	12 (23.1%)	0.911
Family history of diabetes, n (%)	10 (13.7%)	9 (17.3%)	0.602
Creatinine, mg/dL	0.90 [0.20]	0.90 [0.20]	0.061
SCORE-2, %	7.25 [7.83]	12.4 ± 6.86	0.029
SBP 24h, mmHg	112 [11.8]	130±9.31	< 0.001
DBP 24h, mmHg	67.4±8.18	75.3±7.59	< 0.001
MBP 24h, mmHg	83.0 [8.75]	93.4±7.90	< 0.001
FC 24h, bpm	76.9±11.5	71.6±9.93	0.011
DS 24h, mmHg	12.2±3.77	13.1 [4.85]	0.036
SBP day, mmHg	118±12.3	133±10.6	< 0.001
DBP day, mmHg	71.0±9.39	78.3±8.77	< 0.001
MBP day, mmHg	87.3±9.51	97.3±8.31	< 0.001
FC day, bpm	80.1±12.2	74.9±10.9	0.023
DS day, mmHg	9.93 [3.22]	11.6 [4.30]	0.549
SBP night, mmHg	103 [10.3]	120±10.1	< 0.001
DBP night, mmHg	58.5 [8.00]	68.5±7.17	< 0.001
MBP night, mmHg	74.0 [6.75]	86.4±7.07	< 0.001
FC night, bpm	69.66±11.48	64.74±10.02	0.022
DS night	8.37±3.44	9.83±3.25	0.029
Dipping, mmHg	11.85±6.27	9.88±7.35	0.138
PP 24h, mmHg	46.33±7.18	53.98±8.39	< 0.001
BPV	8.40±3.43	9.60±3.26	0.076
PWV, m/s	7.51±1.57	9.02±1.81	< 0.001
PWV > 9, n (%)	10 (13.7%)	26 (50.0%)	< 0.001
AO SVd, mm	32.9±4.09	34.2±3.69	0.076
AO STJd, mm	27.7±3.00	28.8±3.32	0.171
AO ASC, mm	33.1 [4.79]	33.8±3.94	0.686
LV Asd, mm	1.01±0.16	1.10±0.18	0.004
LV IDd, mm	4.49±0.55	4.63±0.55	0.179
LV ILd, mm	0.94±0.16	0.98 [0.20]	0.040

LV IDs, mm	30.7±4.80	31.5±4.94	0.373
RWT, mm	0.42±0.09	0.44±0.10	0.323
LVM, g	141 [49.4]	175 [64.1]	0.004
LVMi, g/m2	78.1±23.8	94.7±30.2	0.011
LVH, %	17 (23.3%)	25 (48.1%)	0.005
LVEDV, mL	87.9±28.1	86.5±31.4	0.330
LVEDVi, mL/m2	49.8±15.0	51.1±13.3	0.624
LVEF, %	61.7±6.23	61.9±4.72	0.653
GLS, %	-21.9±2.32	-21.7±2.74	0.601
GLS <20, n (%)	11 (15.1%)	13 (25.0%)	0.264
TAPSE, mm	24.3 ±4.23	24.6 ± 4.56	0.769
TR Vmax, cm/sec	2.20±0.39	2.37±0.43	0.072
PAPS, mmHg	21.4±7.15	25.5±8.13	0.022
TM A, cm/sec	0.67 ±0.16	0.73 ±0.12	0.033
TM E, cm/sec	0.61 [0.226]	0.64 ±0.15	0.897
E/A	0.98 ±0.30	0.82 [0.31]	0.032
LtdiE, cm/sec	9.42±2.47	8.19 [2.82]	0.016
StdiE, cm/sec	7.34±1.73	5.95 [1.61]	< 0.001
LtdiS, cm/sec	9.26±2.09	9.42±2.34	0.698
StdiS, cm/sec	7.51±1.27	7.34±1.43	0.497
MtdiE, cm/sec	8.36±1.85	6.97 [1.45]	< 0.001
E/e′	7.38 [2.74]	9.05 ±2.53	0.009
LAS reservoir, %	40.5±12.1	38.1±11.2	0.260
LAS conduit, %	-21.0 ± 7.59	-15.1 [8.32]	0.002
LAS contractile, %	-19.9 ± 7.16	-21.6 ± 7.58	0.225
LAVi, mL/m2	26.4 [13.0]	29.5±7.60	0.577
LAe, n (%)	18 (24.7%)	11 (21.1%)	0.608

BSA: Body Surface Area, BMI: Body Mass Index, SBP: Systolic Blood Pressure, DBP: Diastolic Blood Pressure, SCORE-2: Systematic COronary Risk Evaluation, DS day: Standard Deviarion Daytime Blood Pressure, BPV: Blood Pressure Variability, PWV: Pulse Wave Velocity, AO ASC: Ascending Aorta diameter, LVM: Left Ventricular Mass, LVMi: Left Ventricular Mass index, LVH: Left Ventricular Hypertrophy, LVEDV: Left Ventricular End-Diastolic Volume, LVEDVi: Left Ventricular End-Diastolic Volume index, LVEF: Left Ventricular Ejection Fraction, GLS: Global Longitudinal Strain, TAPSE: Tricuspid Annular Plane Systolic Excursion, LAS: Left Atrial Strain, LAVi: Left Atrial Volume index, LAe: Left Atrial Enlargement.

Table S2: Continuous variables: univariate analisys.

Covariate	Beta	HR (95% CI for HR)	Wald test	p.value	Cox assumption	N eventi
Systolic Blood Pressure, mmHg	0.02	1.02 (1.01-1.04)	13.25	**<0.001**	0.485	64
BPV	0.08	1.08 (1.01-1.16)	5.70	**0.017**	0.024	55
PWV	0.17	1.19 (1.05-1.35)	7.14	**0.008**	0.186	60
AO ASC	0.02	1.02 (0.97-1.07)	0.38	0.538	0.101	50
LVEDVi	-0.00	1.00 (0.98-1.01)	0.23	0.629	0.326	62
GLS	0.10	1.10 (1.00-1.22)	3.49	0.062	0.422	59
LAVi	0.01	1.01 (0.99-1.04)	0.95	0.329	0.228	62
LVEF	0.00	1.00 (0.96-1.05)	0.04	0.837	0.928	64
LAS conduit	-0.04	0.96 (0.93-1.00)	4.83	**0.028**	0.334	64

BPV: blood pressure variability, PWV: pulse wave velocity, AO ASC: ascending aorta diameter, LVEDVi: left ventricular end diastolic volume index, GLS: global longitudinal strain, LAVi: left atrial volume index, LVEF: left ventricular ejection fraction, LAS: left atrial strain.

Table S3: Continuous variables: multivariate analisys.

Covariate	Beta	HR (95% CI for HR)	p value	Covariate	Beta	HR (95% CI for HR)
Systolic Blood Pressure, mmHg	0.02	1.02 (1.00-1.03)	**0.049**	PAS semiorto, mmHg	0.02	1.02 (1.00-1.03)
BPV	0.05	1.05 (0.97-1.14)	0.208	BPV	0.05	1.05 (0.97-1.14)
PWV, m/s	0.03	1.03 (0.88-1.20)	0.736	PWV, m/s	0.03	1.03 (0.88-1.20)
LAS conduit, %	-0.04	0.96 (0.92-1.00)	**0.045**	LAS conduit, %	-0.04	0.96 (0.92-1.00)

BPV: blood pressure variability, PWV: pulse wave velocity, LAS: left atrial strain

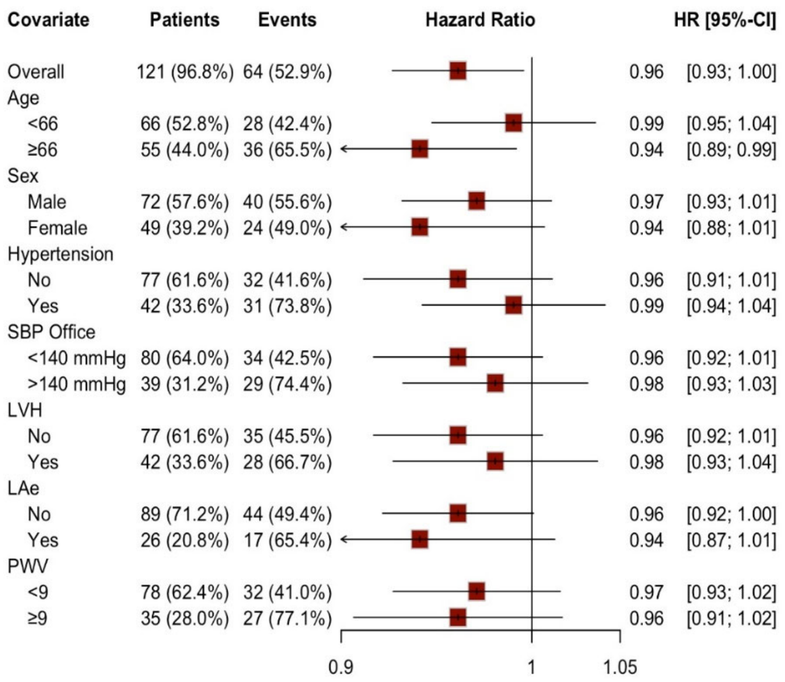

Figure S2: Forest plot: LAS-cd as a predictive factor in different sub-population. *SBP: Systolic blood pressure, LVH: Left ventricular hypertrophy, LAe: Left Atrial Enlargement, PWV: Pulse Wave Velocity*
